# 15-F_2t_-Isoprostane Favors an Anti-Inflammatory Phenotype in RAW 264.7 Macrophages during Endotoxin Challenge

**DOI:** 10.3390/antiox11030586

**Published:** 2022-03-19

**Authors:** Ashley K. Putman, G. Andres Contreras

**Affiliations:** Department of Large Animal Clinical Sciences, College of Veterinary Medicine, Michigan State University, 784 Wilson Road, East Lansing, MI 48823, USA; contre28@msu.edu

**Keywords:** isoprostane, oxidative stress, inflammation, macrophage

## Abstract

Dysregulated inflammation and oxidative stress are major underlying components of several diseases. Macrophages are critical effector cells in immune responses, functioning to progress and resolve inflammation during such diseases. These mononuclear cells regulate inflammatory responses by exhibiting a range of phenotypes that evolve with the process, first promoting inflammation but then switching to a proresolving subtype to restore tissue homeostasis. Furthermore, macrophages are a primary source of isoprostanes (IsoPs), a nonenzymatic byproduct of lipid peroxidation during inflammation. As highly sensitive and specific indicators of lipid damage, IsoPs are the gold standard biomarker of oxidative stress. However, the physiological role of IsoPs during inflammation is currently not well-established. This study determined how IsoPs affect macrophage phenotype during lipopolysaccharide (LPS) challenge. RAW 264.7 macrophages (*n* = 7) were challenged with 5 ng/mL LPS for 8 h, followed with or without 500 nM 15-F_2t_-IsoP for 1 h. Macrophage phenotype was determined using metabolic, transcriptomic, and proteomic markers. Phenotypic markers assessed included ATP production; transcription of proinflammatory *Nos2*, *Il1β*, and anti-inflammatory *Il10*; and translation markers IL1α and IL6 (proinflammatory) with G-CSF and IL17 (anti-inflammatory). Statistical analyses included one-way ANOVA followed by Tukey’s posthoc test. Significance was set at *p* < 0.05. In combination with LPS, 15-F_2t_-IsoP increased ATP production relative to LPS-only treated cells. Additionally, gene expression of *Nos2* and *Il1β* were decreased while *Il10* was increased. Cytokine production of IL6 was decreased while IL10, G-CSF, and IL17 were increased. Collectively, these results provide evidence that 15-F_2t_-IsoP promotes an anti-inflammatory macrophage phenotype during LPS challenge. These data support a novel physiological role of IsoPs, where these lipid mediators may participate in healing pathways during late-stage inflammation when they are elevated. Additionally, the promotion of an anti-inflammatory macrophage phenotype may contribute to preventing or mitigating inflammation during disease. Future studies should be directed towards defining the mechanisms in which IsoPs influence macrophage phenotype, such as receptor interactions and downstream signaling pathways.

## 1. Introduction

Oxidative stress is a recognized underlying component of numerous pathologies including cardiovascular disease, neurodegenerative disorders, and diabetes [1]. Oxidative stress occurs when chemically reactive oxygen and nitrogen species (ROS and RNS, respectively) overwhelm antioxidant defenses, leading to damage of host DNA, proteins, and lipids [2]. At amounts that are well-balanced by antioxidant mechanisms, reactive metabolites participate in many homeostatic immune functions, such as phagocytosis and inflammatory signaling pathways [3]. However, increased reactive metabolites during times of oxidative stress contribute to sustained dysregulated inflammation. For example, ROS damage to lipid membranes causes a loss of cellular function leading to impaired immunity, which ultimately predisposes individuals to increased disease susceptibility [3,4]. Macrophages are critical effector cells in immune responses, which can contribute to or be influenced by oxidative stress and dysregulated inflammatory responses.

Macrophages are active participants in promoting and resolving inflammation. Upon stimulation with bacterial components, such as lipopolysaccharide (LPS), macrophages are activated to a proinflammatory phenotype [5]. Initiating this reaction during endotoxin challenge is necessary to neutralize the inciting cause. As inflammation progresses, an anti-inflammatory phenotype must be acquired to promote resolution and tissue healing [6]. Traditionally, the proinflammatory phenotype is associated with expression of nitric oxide synthase 2 (*Nos2*) and cytokines such as interleukin (IL) 1β and IL6 [5]. It is also well-established that macrophages stimulated with LPS undergo a metabolic switch from oxidative phosphorylation to aerobic glycolysis [7]. By converting to glycolysis, these cells are primed for the rapid ATP, ROS, and cytokine production necessary for host defense [8,9]. On the other hand, macrophages polarized to the alternatively-activated phenotype are generally associated with anti-inflammatory effects. Expression of markers such as *Il10* and arginase 1 (*Arg1*), along with energy production via oxidative phosphorylation, characterize this subtype of macrophage [5]. It is suggested that these changes may be beneficial in promoting cell survival and tissue repair rather than being optimized for pathogen-killing, as are glycolysis-utilizing cells [7]. Regardless of phenotype, macrophages are primary producers of ROS. For instance, ROS are produced by macrophages as a part of the oxidative burst during phagocytosis or from the mitochondria during innate immune responses [10]. The close proximity of generated ROS and cellular lipid membranes puts macrophages in an optimal position to generate a certain byproduct of oxidative damage known as isoprostanes (IsoPs).

Isoprostanes are produced via a nonenzymatic mechanism when reactive metabolites interact with polyunsaturated fatty acids in lipid membranes [11]. Due to this mechanism of formation, IsoPs are currently considered highly sensitive and specific biomarkers of oxidative stress in vivo [12]. Amongst the hundreds of isomers that can theoretically be generated, the arachidonic-acid-derived 15-F_2t_-IsoP is abundant in mammals and is particularly well-cited in the literature [13,14]. In addition to their role as excellent biomarkers, past studies support that IsoP can influence macrophages during inflammation. For instance, Kumar et al. [15] found that 15-F_2t_-IsoP decreased monocyte adhesion to human dermal endothelial cells challenged with tumor necrosis factor alpha (TNFα). Alterations in macrophage cytokine expression are also possible. Scholz and colleagues [16] demonstrated increased IL8 expression in cells treated with 15-F_2t_-IsoP. Since IsoP synthesis is enhanced as the inflammatory process evolves, these lipid mediators may play a key role in modulating later stages of inflammation. However, the ability of IsoP to alter macrophages towards a pro- or anti-inflammatory phenotype remains poorly characterized. Thus, the objective of this study is to define the effect of 15-F_2t_-IsoP on macrophage polarization during endotoxin challenge.

## 2. Materials and Methods

### 2.1. Reagents

Fetal bovine serum (FBS) was provided by Hyclone Laboratories, Inc. (Logan, UT, USA). The antibiotics/antimycotic solution and L-glutamine were obtained from Life Technologies (Carlsbad, CA, USA). Sodium selenite and the known inducer of apoptosis in RAW cells, staurosporine, were purchased from Sigma-Aldrich (St. Louis, MO, USA). RPMI 1640 media was bought from Cellgro (Manassas, VA, USA). Lipopolysaccharide purified from *E. coli* 0111:B4 was purchased from Invivogen (San Diego, CA, USA). The arachidonic-acid-derived IsoP, 15-F_2t_-IsoP (formerly 8-iso-PGF_2α_), was bought from Cayman Chemical (Ann Arbor, MI, USA).

### 2.2. Cell Culture

RAW 264.7 macrophages were obtained from the American Type Culture Collection (TIB-71; American Type Culture Collection, Manassas, VA, USA). Cells between passages 9 and 45 were grown to 75–80% confluency in flasks incubated at 5% CO_2_ and 37 °C. Media for the RAW 264.7 cells consisted of RPMI 1640 containing 5% FBS, 100 U/mL antibiotics and antimycotics, 300 mg/mL L-glutamine, and 0.1 μM sodium selenite.

### 2.3. Experimental Design

**Figure 1:**
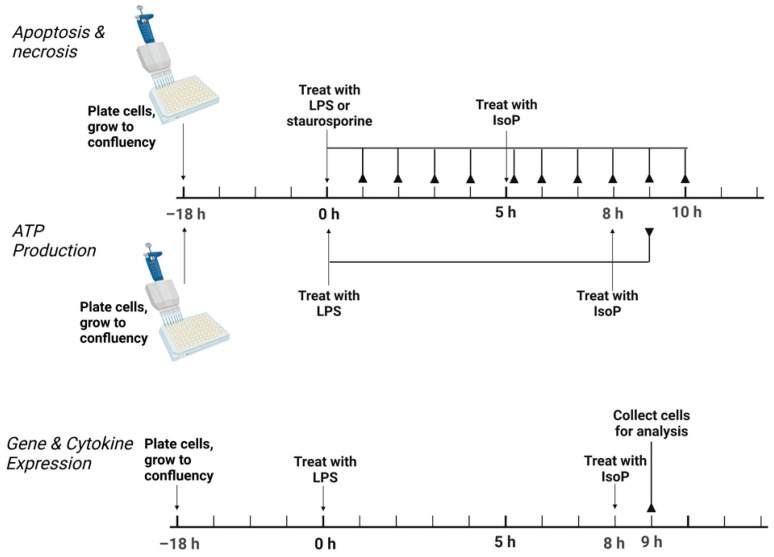
Experimental design to test the effect of 15F_2t_isoprostane on RAW 264.7 macrophage polarization. For apoptosis, necrosis, and ATP production, the solid lines ending in reverse arrowheads represent timepoints when plates were read on a Tecan Infinite 200 Pro. For gene and cytokine expression, the solid line ending in a reverse arrowhead represents the point at which cells were collected for further analysis. LPS—lipopolysaccharide; IsoP—isoprostane; h—hour.

Conditions of acute inflammation were generated with LPS. Following plating, cells were allowed to grow to confluency for 18 h. In general, treatments consisted of the following: untreated media control, vehicle control with 0.05% by volume ethanol, 5 ng/mL LPS, 500 nM 15-F_2t_-IsoP, and 5 ng/mL LPS + 500 nM 15-F_2t_-IsoP. For assessment of apoptosis, an additional treatment of 1000 nM staurosporine was included as a positive control. For all assays except for apoptosis, LPS was added to cell culture media and incubated for 8 h, followed by the addition of IsoP for 1 h. For the apoptosis assay, cells were incubated for 5 h after the addition of LPS or staurosporine before the addition of IsoP for 1 h. A graphical summary of the experimental design is presented in Figure 1.

### 2.4. Apoptosis and Necrosis

To ensure that both the dose of LPS and IsoP used in the present study were not causing apoptosis and subsequent necrosis in the RAW 264.7 cells, a Promega RealTime-Glo Annexin V Apoptosis and Necrosis Assay (Madison, WI, USA) was employed. Briefly, this assay measures the amount of phosphatidylserine present on the outer leaflet of cell membranes. An increase in luminescence is detected when a greater amount of phosphatidylserine is exposed on the outer leaflet, which occurs during early apoptosis. As membrane integrity decreases with the progression of apoptosis to necrosis, a DNA-binding dye generates a fluorescent signal.

Cells were seeded in white, flat-bottom, 96-well plates at a density of 4.0 × 10^4^/well; then, the protocol was carried out following the instructions provided in the kit. Using a Tecan Infinite 200 Pro (Männedorf, Switzerland), luminescence was read along with fluorescence at 485 nm excitation and 525 nm emission.

### 2.5. ATP Production

The production of ATP was assessed with Promega CellTiter-Glo Luminescent Cell Viability Assay. This assay produces a luminescent signal proportional to the amount of ATP present in a well. Cells were plated in Costar white, flat-bottom, 96-well plates at a density of 4.0 × 10^4^/well. The assay was performed according to manufacturer’s instructions. Luminescence was read on a Tecan Infinite 200 Pro. Doses of 10–500 nM IsoP were used to establish the optimal concentration to produce a maximal alteration of ATP production in macrophages relative to LPS treatment.

### 2.6. Gene Expression

RAW 264.7 cells were cultured in 6-well plates for RNA extraction. Wells were seeded at 1.0 × 10^6^ cells and grown to 75–80% confluency. After incubation, following the treatments described above, each well was washed twice with HBSS followed by 300 μL Buffer RLT (Qiagen, Hilden, Germany) to lyse the cells. The buffer was collected from each well and then added to a 1.5 mL microcentrifuge tube. Cell lysate was stored at −20 °C pending RNA extraction within 1 mo of collection.

Extraction of RNA occurred utilizing a Promega Maxwell RSC Instrument following the manufacturer’s protocol. Quantification and the quality of RNA was assessed with the Nucleid Acid Quantification program (i-control 1.11, Tecan, Männedorf, Switzerland) using a NanoQuant plate read on a Tecan Infinite 200 Pro. RNA was then stored at −20 °C until cDNA was generated.

Prior to cDNA generation, all samples were standardized with nuclease-free water to 100 ng/μL. An equal volume of master mix containing 10× reverse-transcription buffer, 25× dNTP, 10× random primers, Multiscribe reverse transcriptase, RNase inhibitor, and RNase nuclease-free water from a high-capacity cDNA reverse-transcription kit with RNase inhibitor (Applied Biosystems High-Capacity cDNA Archive Kit, Waltham, MA, USA) was added to each standardized RNA sample. Samples were placed in a MiniAmp Plus Thermal Cycler (Applied Biosystems, Waltham, MA, USA) programmed with the following settings: 25 °C for 10 min, followed by 37 °C for 2 h, then 85 °C for 5 min, finishing with 4 °C hold until samples were removed. Samples were then stored at −20 °C until qRT-PCR was completed.

Real-time PCR was carried out with predesigned TaqMan primers and FAM-MGB probes (Applied Biosystems). Samples were assessed for *Nos2* (Mm00440502_m1), *Il1β* (Mm00434228_m1), *Il10* (Mm01288386_m1), *Arg1* (Mm00475988_m1), actin B (*Actb*, endogenous control, Mm02619580_g1), and glyceraldehyde-3-phosphate dehydrogenase (*Gapdh*, endogenous control, Mm99999915_g1). Genes were evaluated in triplicate with 2× TaqMan Gene Expression Master Mix (Applied Biosystems), 20× TaqMan Gene Expression Assay Mix (Applied Biosystems), sample cDNA (50 ng/well), and nuclease-free water for a total of 10 μL per reaction well. Thermal cycling conditions for the Fast 2-step PCR system were as follows: stage 1, 95 °C for 20 s; stage 2, 95 °C for 3 s; stage 3, 60 °C for 30 s, with 40 cycles of stages 2 and 3. Data were recorded and compiled using ExpressionSuite Software version 1.3.

### 2.7. Cytokine and Chemokine Production

Cytokines and chemokines commonly associated with inflammation were evaluated with a Milliplex Mouse Cytokine/Chemokine Magnetic Bead Panel—Premixed 25 Plex—Immunology Multiplex Assay (Millipore Sigma, Burlington, MA, USA). The cytokines and chemokines in this panel included the following: granulocyte colony-stimulating factor (G-CSF); granulocyte-macrophage colony-stimulating factor (GM-CSF); interferon gamma (INFγ); IL1α; IL1β; IL2; IL4; IL5; IL6; IL7; IL9; IL10; IL12p40; IL12p70; IL13; IL15; IL17; interferon gamma-induced protein 10 (IP10); chemokine ligand 1 (alternatively, keratinocyte-derived chemokine—mKC); monocyte chemoattractant protein-1 (MCP-1); macrophage inflammatory protein-1 alpha (MIP-1α); MIP-1β; MIP-2; regulated on activation, normal T cell expressed and secreted (RANTES); TNFα. The assay was performed according to manufacturer’s instructions. Untreated media control, ethanol vehicle control, and 500 nM 15-F_2t_-IsoP samples were not diluted for the assay. However, 5 ng/mL LPS and 5 ng/mL LPS + 500 nM 15-F_2t_-IsoP samples were diluted 1:10 to ensure the concentrations would fall within the dynamic range of the assay. The plate was read with a Luminex 200 System (Austin, TX). The data were analyzed with xPONENT software version 3.1.

### 2.8. Statistical Analysis

Results for apoptosis, necrosis, and cytokine production are presented as least squares means + standard error of the mean. Production of ATP is represented as a ratio of the least squares means + standard error of the mean in treated cells to the untreated media control. The results for PCR were analyzed with the ΔΔCt method and are graphically represented as relative expression (2^−ΔΔCt^) [17]. As the absolute quantification of copy numbers was not necessary to determine how IsoPs may affect gene expression, utilizing this method is advantageous in that it does not require the generation of a standard curve. Sample size was calculated a priori based on unpublished preliminary ATP production data. The PROC POWER function of SAS 9.4 (Cary, NC, USA) was utilized with the following syntax: proc power; onewayanova; groupmeans = 1 (untreated media control), 0.84 (ethanol vehicle control), 0.68 (5 ng/mL LPS), 0.82 (5 ng/mL LPS + 500 nM 15-F_2t_-IsoP); stddev = 0.1; alpha = 0.05; npergroup = .; power = 0.9. Power analysis revealed that a sample size of 4 was sufficient to detect a 1-fold difference in ATP production between treatments. Normality was visually assessed and confirmed with a Shapiro–Wilk test. A linear mixed effects model (variables = time and treatment) and Tukey’s HSD posthoc test were used for apoptosis and necrosis results. ATP production, PCR, and cytokine expression were analyzed with one-way ANOVA and Tukey’s posthoc test. For cytokine production, cytokines with R^2^ < 90% were excluded from analyses (GM-CSF, IL9, IL15, and MIP-2). To account for family-wise error rate when analyzing the cytokine production data, Bonferroni’s correction was used. Therefore, differences in concentrations between treatments were considered if the ANOVA *p* value was less than 0.002 (0.05/25). All statistical analyses were carried out in SAS 9.4.

## 3. Results

### 3.1. 15-F_2t_-IsoP Is not Cytotoxic to RAW 264.7 Cells

**Figure 2:**
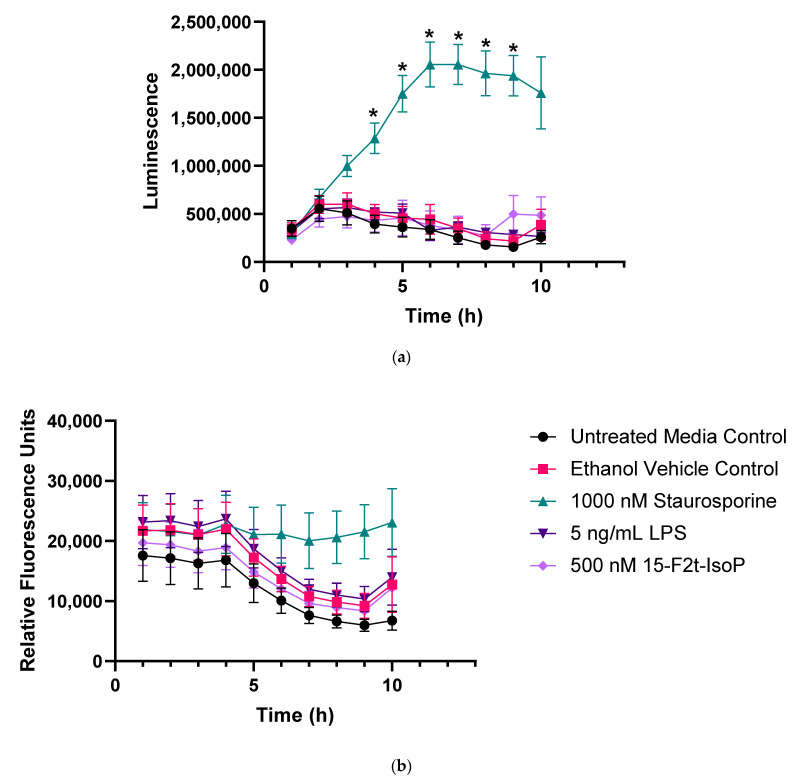
Endotoxin and 15F_2t_-Isoprostane do not cause apoptosis or necrosis in RAW 264.7 macrophages (*n* = 7). (**a**) Mean luminescence, representative of apoptosis; * increased luminescence in the staurosporine treatment group at a given time point (*p* < 0.05). (**b**) Mean fluorescence, resulting from necrosis secondary to apoptosis. Statistical analyses included a linear mixed effects model with Tukey’s HSD posthoc test. LPS—lipopolysaccharide; IsoP—Isoprostane; h—hour.

Figure 2 represents apoptosis and necrosis in untreated and treated RAW cells. Staurosporine-treated cells exhibited increased apoptosis relative to all other treatments between 4 and 9 h (*p* < 0.01). Neither LPS nor 15-F_2t_-IsoP changed apoptosis relative to untreated controls at any time point (*p* = 0.27 and 0.56, respectively; Figure 2a). Although there was an effect of time (*p* < 0.0001) and time × treatment interaction (*p* = 0.006) for necrosis, differences were not detected after correcting for multiple comparisons (Figure 2b). Thus, LPS and 15-F_2t_-IsoP did not cause cell death or secondary necrosis in RAW cells at the doses and incubation times utilized in the present study.

### 3.2. 15-F_2t_-IsoP Increases ATP Production during Endotoxin Challenge

**Figure 3:**
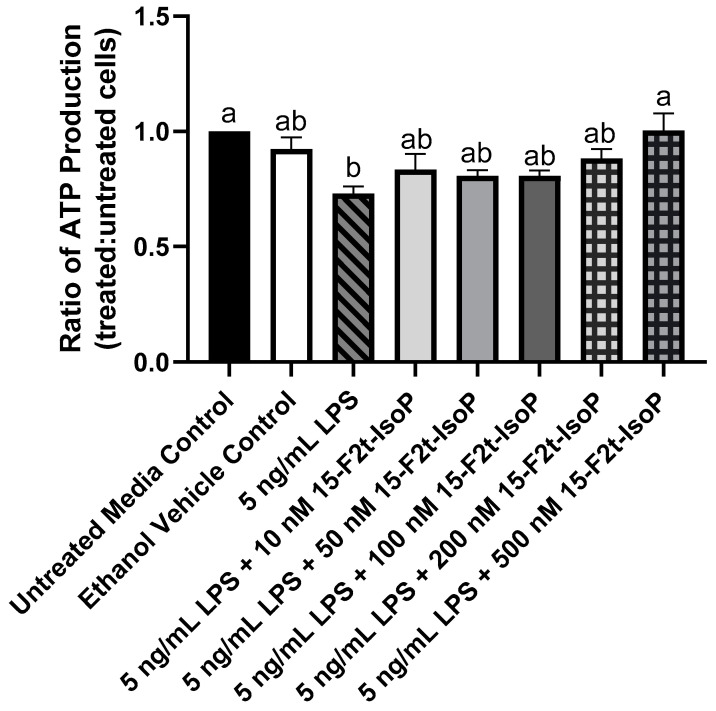
ATP production in RAW 264.7 macrophages (*n* = 7) is altered by 15-F_2t_-isoprostane during endotoxin challenge. Statistical analyses included a one-way ANOVA with Tukey’s HSD posthoc test. ^a,b^ Different superscripts are different (*p* < 0.05). LPS—lipopolysaccharide; IsoP—isoprostane.

During LPS challenge, ATP production was decreased to 73% of that seen in untreated controls (Figure 3; *p* = 0.002). Every treatment that included 10–200 nM 15-F_2t_-IsoP in addition to LPS was not different from LPS only (*p* > 0.26) or untreated controls (*p* > 0.07). Cells treated with LPS + 500 nM 15-F_2t_-IsoP had greater ATP production than cells treated only with LPS (*p* = 0.002). In fact, the LPS + 500 nM 15-F_2t_-IsoP treatment had similar ATP production to untreated controls (*p* > 0.99). As 500 nM IsoP altered ATP production the most compared with LPS alone, it was the concentration utilized for subsequent assays.

### 3.3. 15-F_2t_-IsoP Shifts Inflammatory Gene Expression towards an Anti-Inflammatory Phenotype

**Figure 4:**
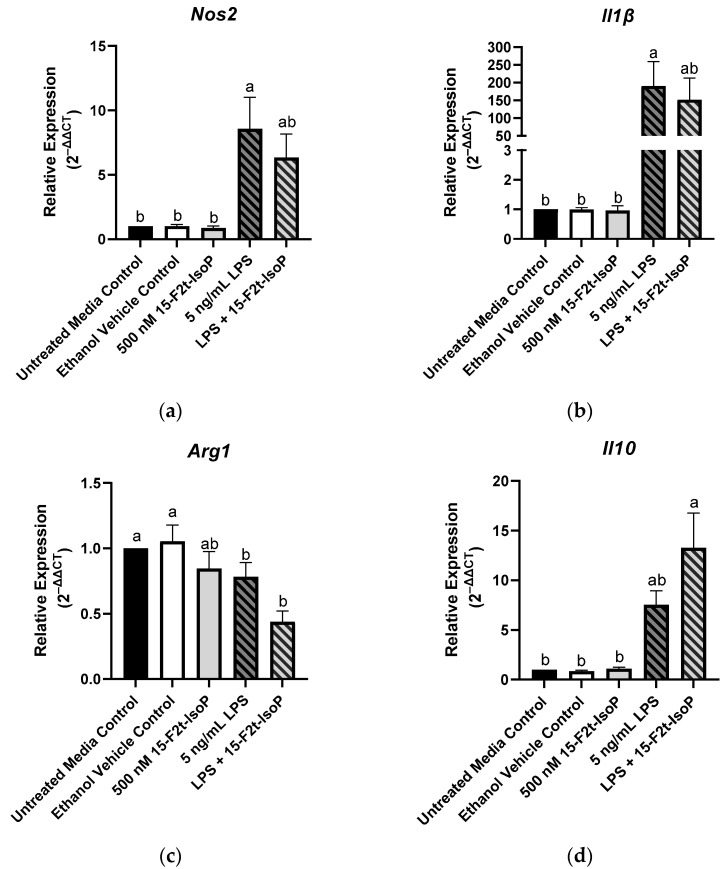
Relative gene expression of common macrophage phenotype markers is shifted in RAW 264.7 cells (*n* = 7) treated with endotoxin and 15-F_2t_-isoprostane: (**a**) nitric oxide synthase 2 (*Nos2*), (**b**) interleukin (*Il*) *1β,* (**c**) arginase 1 (*Arg1*), (**d**) *Il10*. Statistical analyses included a one-way ANOVA with Tukey’s HSD posthoc test. ^a,b^ Different superscripts are different (*p* < 0.05). LPS—lipopolysaccharide; IsoP—isoprostane.

Macrophage phenotype is commonly determined by the relative expression of specific genes. Relative to untreated controls, RAW 264.7 treated with LPS showed increased expression of *Nos2* (2^−ΔΔCt^ = 8.57; *p* = 0.004; Figure 4a) and *Il1β* (2^−ΔΔCt^ = 190; *p* = 0.02; Figure 4b) while relative expression of *Arg1* was decreased to 0.78 (*p* < 0.03; Figure 4c). There was a tendency towards increased *Il10* expression in the LPS-treated cells compared with untreated controls (*p* = 0.08; Figure 4d). When 15-F_2t_-IsoP was added to LPS-treated cells, *Nos2* expression (2^−ΔΔCt^ = 6.34) was not different from untreated controls (*p* = 0.07) or LPS-only treated cells (*p* = 0.78; Figure 4a). Expression of *Il1β* was similarly affected in the LPS and 15-F_2t_-IsoP combination treatment (2^−ΔΔCt^ = 152) compared with untreated and LPS-treated cells. Indeed, LPS + 15-F_2t_-IsoP treatments did not differ from either untreated controls (*p* = 0.1) or LPS treatments (*p* = 0.96; Figure 4b). We did not observe an increase in *Arg1* expression in LPS + 15-F_2t_-IsoP-treated cells relative to the LPS-treated cells (*p* = 0.37; Figure 4c). Finally, the greatest relative expression of *Il10* was observed in cells treated with both LPS and 15-F_2t_-IsoP (2^−ΔΔCt^ = 13.3; Figure 4d). This is compared with relative expression values of 7.5 in LPS-treated RAW cells (*p* = 0.17) and 1 in untreated controls (*p* < 0.0001).

### 3.4. 15-F_2t_-IsoP Alters Cytokine Production Consistent with an Anti-Inflammatory Phenotype

Similar to inflammatory gene expression, production of certain cytokines can indicate macrophage phenotype. The presence of IL4 and IL7 was not detected in any treatment group, while concentrations of MIP-1β were consistently outside of the assay’s limit of detection. Several outliers were detected for IL2; therefore, it was excluded from the study. Table 1 includes the mean concentrations of each remaining cytokine.

**Figure 5:**
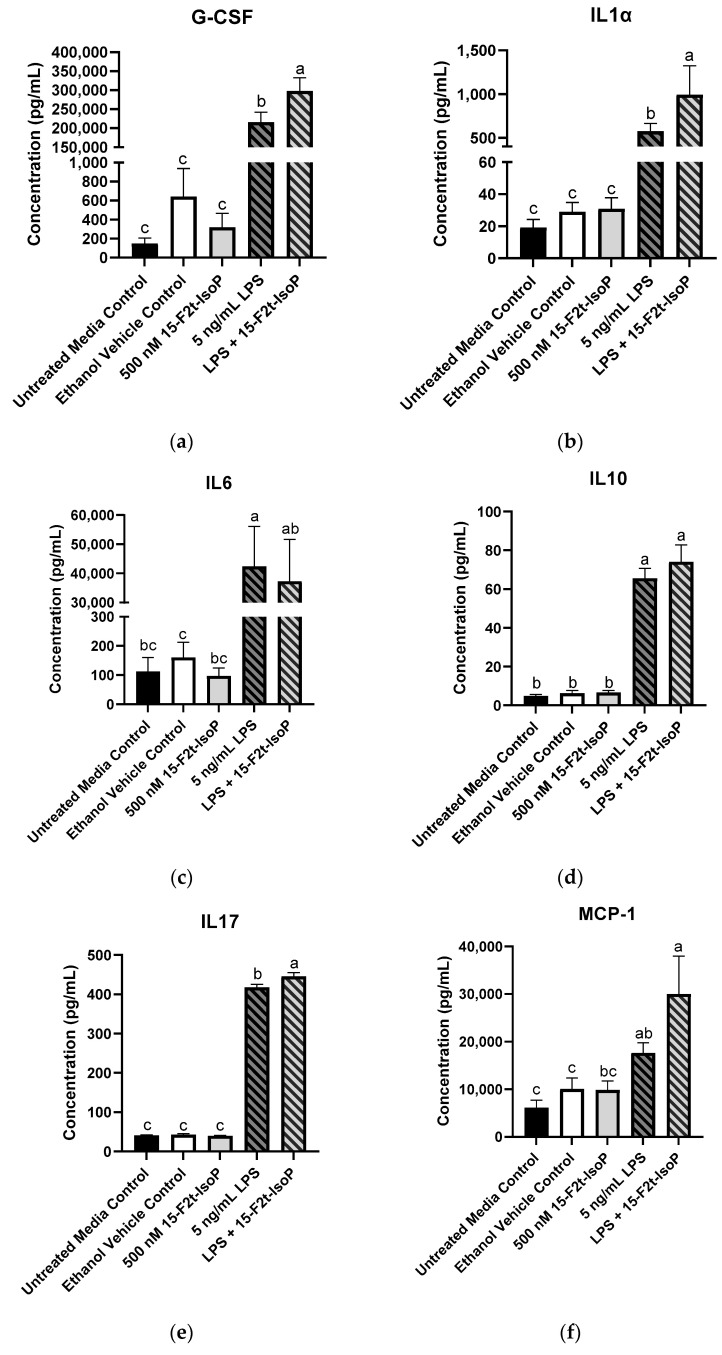
Mean cytokine concentrations in RAW 264.7 cells (*n* = 7) challenged with endotoxin and 15-F_2t_-isoprostane. (**a**) Granulocyte colony-stimulating factor (G-CSF), (**b**) interleukin (IL) 1α, (**c**) IL6, (**d**) IL10, (**e**) IL17, (**f**) monocyte chemoattractant protein-1 (MCP-1). Statistical analyses included a one-way ANOVA with Tukey’s HSD posthoc test. ^a–c^ Different superscripts are different (*p* < 0.05); LPS—lipopolysaccharide; IsoP—isoprostane.

The greatest production of G-CSF (297,845 pg/mL) was seen in 5 ng/mL LPS + 500 nM 15-F_2t_-IsoP-treated cells, which was greater than all other treatments (*p* < 0.03; Figure 5a). Similarly, the LPS and IsoP combination treatment had the greatest concentrations of IL1α compared with all other treatment groups (993 pg/mL; *p* < 0.03; Figure 5b). Concentrations of IL6 were 42,369 pg/mL in LPS-treated cells, which was more production than every treatment (*p* < 0.02) except LPS + 15-F_2t_-IsoP (37,268 pg/mL; *p* = 0.99). Although IL6 concentrations of LPS + 15-F_2t_-IsoP were not different from LPS-only treated cells, they were also not different from untreated media controls (*p* = 0.06; Figure 5c). Production of IL10 (74 pg/mL) was greatest in RAW 264.7 cells treated with both LPS and 15-F_2t_-IsoP. These concentrations were greater than untreated media controls (4.82 pg/mL; *p* < 0.0001) and numerically greater than 5 ng/mL LPS concentrations (66 pg/mL; *p* = 0.65; Figure 5d). Similarly, the 5 ng/mL LPS + 500 nM 15-F_2t_-IsoP treatment had the greatest mean IL17 concentrations of 446 pg/mL. The mean concentration of IL17 in cells treated with LPS only was lower (418 pg/mL; *p* = 0.008), while the mean concentration in untreated media controls was even lower still (41 pg/mL; *p* < 0.0001; Figure 5e). Concentrations of MCP-1 were 30,013 pg/mL in the LPS and IsoP combination treatment, which were greater than every other treatment except LPS only (17,655 pg/mL; *p* = 0.21; Figure 5f). The production of these cytokines across treatments have been highlighted in Figure 5.

For the following cytokines, concentrations were increased in cells treated with 5 ng/mL LPS compared with untreated media controls: INFγ, IL5, IL12p70, IL13, mKC, MIP-1α, and RANTES (*p* < 0.05; Table 1). Concentrations of the aforementioned cytokines were also increased in LPS + 15-F_2t_-IsoP treatments compared with untreated cells (*p* < 0.05); however, concentrations were not less than LPS-treated cells (*p* > 0.5).

## 4. Discussion

This study provides evidence that 15-F_2t_-IsoP shifts RAW 264.7 macrophages towards an anti-inflammatory phenotype during LPS challenge. Altering macrophage subtype as inflammation proceeds represents a link between increased IsoP formation during oxidative stress and inflammatory outcomes.

Metabolic parameters, such as ATP production, can be used to help distinguish macrophage phenotypes [18]. It is well-established that LPS shifts macrophage metabolism from oxidative phosphorylation to aerobic glycolysis, thus preparing proinflammatory cells for host defense. However, while glycolysis produces ATP rapidly, it does so inefficiently [7]. Therefore, LPS-stimulated, proinflammatory macrophages generate less ATP compared with the anti-inflammatory subtype [18]. Thus, our findings that LPS decreases ATP production without a concurrent increase in cell death agree with several other studies [19,20,21]. Furthermore, the finding that adding 15-F_2t_-IsoP to LPS-treated cells returns ATP production to amounts similar to unstimulated macrophages supports that the lipid mediator may promote a metabolic switch back to oxidative phosphorylation. As cells performing oxidative phosphorylation are well-positioned for inflammatory resolution, IsoP may be fostering anti-inflammatory responses during endotoxin challenge [5].

The metabolic switch that occurs in macrophages is also associated with altered gene transcription. In particular, *Nos2* and *Il1β* are commonly reported for proinflammatory macrophages while *Il10* and *Arg1* are considered landmark genes for the anti-inflammatory subtype [22]. Indeed, increased *Nos2* expression is paramount in the phenotypic switch towards a proinflammatory state, partially because of the subsequent changes in mitochondrial respiration and ROS production [23]. In our study, RAW 264.7 cells treated with both LPS and IsoP showed a numerical decrease in *Nos2* expression relative to the LPS treatment. Therefore, by decreasing *Nos2* (and, theoretically, ROS production), 15-F_2t_-IsoP may be capable of antioxidant activity. Musiek et al. [24] described decreased Nos2 protein expression and activity in RAW cells treated with 15-A_2_-IsoP, an isomer that contains a cyclopentenone ring considered responsible for this bioactivity. However, the authors did not see a similar reduction in Nos2 activity when cells were treated with 15-F_2t_-IsoP [24]. Thus, characterizing the mechanisms behind which 15-F_2t_-IsoP caused a relative decrease in *Nos2* gene expression and the functional consequences of these changes is warranted.

In addition to the alterations in *Nos2*, the transcriptional signature of cells treated with 15-F_2t_-IsoP and LPS were suggestive of an anti-inflammatory macrophage phenotype. For instance, there was a relative decrease in *Il1β* with a concurrent relative increase in *Il10* compared with the cells challenged with only LPS. Transcription of *Il1β* is associated with increased inflammation while *Il10* is able to decrease glycolysis, effectively inhibiting proinflammatory macrophage function [25,26]. Hence, IsoP may be supporting the anti-inflammatory macrophage subtype by decreasing expression of these genes. We also observed an expected decrease in *Arg1* expression from LPS-treated RAW cells. Interestingly, however, the downregulation was even more pronounced in the LPS and IsoP combination treatment. This is in contrast with previous work demonstrating an increase in *Arg1* expression in alternatively-activated cells [5,27,28]. However, macrophage gene expression can be complex, falling outside the traditional polarization dichotomy [29,30]. Thus, similar to conditions found in tissues under oxidative stress, it is likely that the polarization of macrophages in our model is along a spectrum rather than at either extreme. Further functional and mechanistic characterization of macrophages treated with IsoP are necessary to determine the implications of the gene expression signatures noted herein.

In the face of endotoxin challenge, altered gene regulation leads to the production of cytokines and chemokines meant to appropriately progress and resolve inflammation. Proinflammatory macrophages will secrete proteins that are designed to trigger Th1 responses (e.g., IL6) with the goal of killing pathogens [29]. On the other hand, anti-inflammatory cells will generate cytokines such as IL10 that invoke tissue repair and downregulation of inflammatory responses [31]. Several cytokines associated with proresolving functions were upregulated in RAW cells treated with both LPS and 15-F_2t_-IsoP presently. These included G-CSF, IL10, and IL17. Furthermore, there were numerical decreases in the proinflammatory IL6 compared with LPS-treated cells. Taken together, these data support an anti-inflammatory phenotype predominated when IsoP was added to LPS-treated cells. Indeed, G-CSF is a well-established inducer of alternatively-activated macrophages and IL17 has many protective effects during inflammation, such as initiating a negative feedback loop to downregulate nuclear factor kappa b activity [32,33]. Additionally, IL1α and MCP-1 were also upregulated in LPS + IsoP-treated cells. Although IL1α is commonly associated with proinflammatory effects, it is unclear at this time how the increased concentrations seen with LPS + IsoP treatment may influence inflammatory outcomes [34]. Several studies indicate that MCP-1 potently promotes Th2 responses and can therefore be associated with anti-inflammatory functions [35]. Furthermore, F2-IsoPs were positively correlated with MCP-1 expression in chronic obstructive pulmonary disease [36]. Thus, there may be a mechanism in which elevated IsoPs contribute to increased MCP-1 expression to mitigate oxidative stress and inflammation as the processes progress.

## 5. Conclusions

This study demonstrates a novel physiological role of 15-F_2t_-IsoP, where this lipid mediator may participate in healing pathways during late-stage inflammation when they are elevated. Thus, this study supports that 15-F_2t_-IsoPs serve as a link between oxidative stress and inflammation. Future studies should be directed towards determining specific mechanisms in which this isomer influences macrophages, such as the receptor they are acting through and downstream signaling pathways. It would also be beneficial to investigate functional consequences of the IsoP-mediated switch to an anti-inflammatory phenotype.

## Figures and Tables

**Table 1:** Mean concentrations (pg/mL) of cytokines produced by RAW 264.7 cells (*n* = 7) treated with endotoxin and 15-F_2t_-isoprostane. Statistical analyses included a one-way ANOVA with Tukey’s HSD posthoc test unless indicated otherwise. Bonferroni’s correction was used to account for family-wise error (*p* < 0.002). ^a–c^ Means within a row with different superscripts are different (*p* < 0.05). LPS—lipopolysaccharide; G-CSF—granulocyte colony-stimulating factor; INF—interferon; IL—interleukin; IP—interferon gamma-induced protein; mKC—chemokine ligand 1; MCP—monocyte chemoattractant protein; MIP—macrophage inflammatory protein; RANTES—regulated on activation, normal T cell expressed and secreted; SD—standard deviation; *—Kruskal–Wallis analysis (*p* < 0.05), but no significant differences after correcting for multiple comparisons with Dunn’s test.

	Untreated Media Control	Ethanol Vehicle Control	500 nM 15-F_2t_-IsoP	5 ng/mL LPS	LPS + 15-F_2t_-IsoP	Pooled SD	*p*
G-CSF	149 ^c^	642 ^c^	320 ^c^	216,025 ^b^	297,845 ^a^	45,800	<0.0001
INFγ	2.42 ^b^	5.41^b^	3 ^b^	94.1 ^a^	97 ^a^	22.1	<0.0001
IL1α	19.2 ^c^	29 ^c^	30.8 ^c^	575 ^b^	993 ^a^	190	<0.0001
IL1β	3	3	3	3	8.35	4.41	0.38
IL5	12.3 ^b^	14.1 ^b^	13.1 ^b^	91.1 ^a^	92.1 ^a^	9.89	<0.0001
IL6	112 ^bc^	161 ^c^	97 ^c^	42,369 ^a^	37,268 ^ab^	21,400	0.001
IL10	4.82 ^b^	6.22 ^b^	6.59 ^b^	65.5 ^a^	74 ^a^	10.8	<0.0001
IL12p40 *	4.49	12.4	7.19	21.9	3	20.4	0.14
IL12p70	3 ^b^	3 ^b^	58.8 ^a^	3 ^b^	38.6 ^a^	17.5	<0.0001
IL13	38.3 ^b^	21.1 ^b^	20.6 ^b^	958 ^a^	894 ^a^	257	<0.0001
IL17	41.5 ^c^	42.9 ^c^	40 ^c^	418 ^b^	446 ^a^	13.2	<0.0001
IP10	8.42	7.43	10.8	63.1	94.3	75.3	0.22
mKC	3 ^b^	3 ^b^	2.66 ^b^	59.8 ^a^	50.1 ^a^	15.8	<0.0001
MCP-1	6113 ^c^	10,066 ^c^	9840 ^bc^	17,655 ^ab^	30,013 ^a^	9740	0.002
MIP-1α	3109 ^b^	4413 ^b^	4652 ^b^	133,077 ^a^	155,781 ^a^	35,900	<0.0001
RANTES	4.3 ^b^	4.41 ^b^	4.58 ^b^	98.8 ^a^	146 ^a^	57.6	0.0001
TNFα *	240	209	222	66,800	10,001	34,900	0.005

## Data Availability

Data sharing not applicable.
